# Color Photographic Index of Fall Chinook Salmon Embryonic Development and Accumulated Thermal Units

**DOI:** 10.1371/journal.pone.0011877

**Published:** 2010-07-29

**Authors:** James W. Boyd, Eric W. Oldenburg, Geoffrey A. McMichael

**Affiliations:** Ecology Group, Battelle, Richland, Washington, United States of America; University of Birmingham, United Kingdom

## Abstract

**Background:**

Knowledge of the relationship between accumulated thermal units and developmental stages of Chinook salmon embryos can be used to determine the approximate date of egg fertilization in natural redds, thus providing insight into oviposition timing of wild salmonids. However, few studies have documented time to different developmental stages of embryonic Chinook salmon and no reference color photographs are available. The objectives of this study were to construct an index relating developmental stages of hatchery-reared fall Chinook salmon embryos to time and temperature (e.g., degree days) and provide high-quality color photographs of each identified developmental stage.

**Methodology/Principal Findings:**

Fall Chinook salmon eggs were fertilized in a hatchery environment and sampled approximately every 72 h post-fertilization until 50% hatch. Known embryonic developmental features described for sockeye salmon were used to describe development of Chinook salmon embryos. A thermal sums model was used to describe the relationship between embryonic development rate and water temperature. Mean water temperature was 8.0°C (range; 3.9–11.7°C) during the study period. Nineteen stages of embryonic development were identified for fall Chinook salmon; two stages in the cleavage phase, one stage in the gastrulation phase, and sixteen stages in the organogenesis phase. The thermal sums model used in this study provided similar estimates of fall Chinook salmon embryonic development rate in water temperatures varying from 3.9–11.7°C (mean = 8°C) to those from several other studies rearing embryos in constant 8°C water temperature.

**Conclusions/Significance:**

The developmental index provides a reasonable description of timing to known developmental stages of Chinook salmon embryos and was useful in determining developmental stages of wild fall Chinook salmon embryos excavated from redds in the Columbia River. This index should prove useful to other researchers who wish to approximate fertilization dates of Chinook salmon eggs from natural redds, assuming the thermal history of embryos is known.

## Introduction

The relationship between temperature and rate of embryonic development of fish has traditionally been of interest to hatchery managers for estimating time to fry emergence [1]. The general relationship between the rate of development of embryonic fish and temperature was demonstrated as early as 1868 [2]. The rate of embryonic fish development is largely dependent on temperature [3], [4] and has been intensively studied among salmonids [5], [6], [7], [8], [9], [10]. Rate of development typically increases with increasing water temperature, up to species-specific upper thermal limits [4]. Thus, time to reach any developmental stage generally decreases with increasing water temperature.

The temperature-development rate relationship has been accurately described by power law models [9] and generally assumes a sigmoidal shape over a wide temperature range [11]. Over a narrower range of temperatures surrounding the inflection point of the sigmoid curve, a simpler thermal sums model may be as accurate as more complex models for describing the temperature-development rate relationship [7]. The thermal sums model assumes that the time required to reach any developmental stage or to hatch is dependent on a specific amount of accumulated thermal units (e.g., degree days) [8]; [9], [11]. Degree day is defined as the mean temperature, above 0°C, for a given day.

Embryonic development rates vary among salmonid species reared at similar water temperatures [12]. For example, time to 50% hatch varied from 44 to 63 d for five Pacific salmon species reared in constant 10°C water [12]. Although research has documented time to varying developmental stages of sockeye salmon *Oncorhynchus nerka* embryos [13], published information documenting development rates of Chinook salmon *O. tshawytscha* embryos to known stages is scarce [12]. In addition, high-quality color photographs of developmental stages of Chinook salmon embryos are absent in the scientific literature.

Recently, knowledge of the temperature-development rate relationship has stimulated interest among fisheries managers seeking insight into oviposition timing of wild salmonids. In 2008, the Grant County Public Utility District Number 2 (Washington) initiated research investigating the influence of flow fluctuations on fall Chinook salmon redds constructed at high elevations (i.e., redds constructed above an elevation that was protected by flow management guidelines) in the Columbia River. It was unknown how many eggs were deposited in high-elevation redds. Egg quantification occurred in excavated high-elevation redds to estimate maximum loss resulting from periodic dewatering; however, it was unknown whether female Chinook salmon had completed egg deposition by the time of egg quantification. The investigators postulated that females may have returned to a given redd and deposited more eggs, had the redd not been disturbed to quantify the eggs. It was assumed that eggs fertilized greater than 6 days prior to excavation were in a completed redd, based on previous research [14]. Knowledge regarding the relationship between accumulated thermal units and developmental stages of Chinook salmon embryos was essential in determining the date of egg fertilization and therefore whether redds were completed prior to excavation.

This research was a supplementary effort designed to provide information about timing of Chinook salmon egg deposition, in support of a larger egg quantification study whose results will be published separately. The objectives of this research were to construct an index relating developmental stages of hatchery-reared fall Chinook salmon embryos to time and temperature (e.g., degree days) and provide high-quality color photographs of each identified developmental stage. Results from this study aided in addressing knowledge gaps in fall Chinook salmon embryonic development rates and provided a reference index of development to assist with the estimation of fertilization dates of eggs recovered from natural redds.

## Results

**Figure 1:**
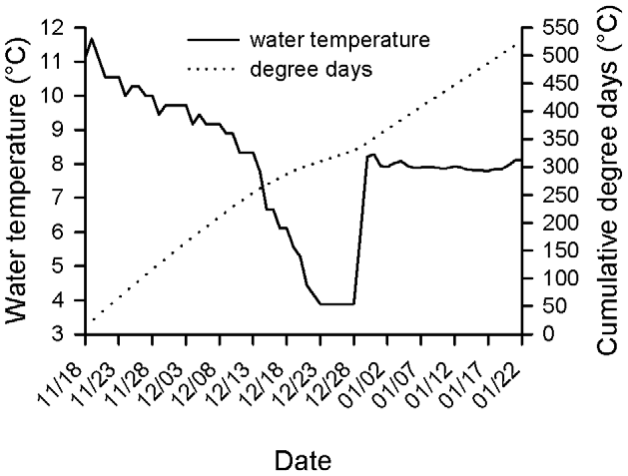
Water temperature and cumulative degree days. Water temperature and cumulative degree days experienced by embryonic fall Chinook salmon during the study period, 2008–2009. Note the increase in water temperature on 29 December 2009 corresponds to the transfer of eggs from Priest Rapids Hatchery to the PNNL Aquatics Research Laboratory.

Mean water temperature was 8.0°C (range; 3.9–11.7°C) during the study period (Figure 1). Nineteen stages of embryonic development were identified for fall Chinook salmon, with each stage representing a discrete snapshot within a time interval when discernable morphological characteristics were present (Table 1). Two stages were observed in the cleavage phase, one stage in the gastrulation phase, and sixteen stages in the organogenesis phase of embryonic development.

**Table 1:** Developmental stages, accumulated temperature and time units, and stage characteristics for fall Chinook salmon embryos.

Stage				
Present study	Velsen (1980)	dd	dpf	Stage characteristics
-	-	0	0	Fertilization
1	8	34	3	Late morula
2	10	66	6	Blastula formation
3	14	96	9	Embryo visible; one-half epiboly; first somites visible; optic vesicles forming
4	18	135	13	Epiboly complete; caudal bud formed and free from yolk surface; lenses in eyes visible
5	19	155	15	Parts of brain distinct; metencephalon developing; vent first visible
6	21	192	19	Vitelline vein first visible; pectoral buds first visible
7	21	211	21	Faint pigment in choroid visible
8	24	237	24	Vascularization branching from vitelline vein; embryo head free from yolk
9	-	261	27	Approximately 50% of choroid pigmented*
10	25	281	30	Anal and caudal fins visible as foggy regions in embryonic finfold
11	-	298	33	Choroid fully pigmented*
12	25	310	36	Anal fin rays forming; operculum present but not covering any branchial arches
13	26	322	39	Operculum partially covering first branchial arch
14	27	336	42	Operculum covers first branchial arch; dorsal fin starting to form
15	28	385	48	Pelvic fin buds first visible; operculum covers part of second branchial arch
16	29	410	51	Operculum covers second branchial arch; caudal fin rays first visible
17	-	435	54	Operculum covers third branchial arch*
18	-	484	60	Operculum partially covers fourth branchial arch*
19	30	533	66	50% hatch

Developmental stage [this study and Velsen (1980)], degree days (dd; °C), days post-fertilization (dpf), and stage characteristics by sample date for hatchery-reared fall Chinook salmon embryos. Asterisks denote characteristics observed in some stages of Chinook salmon development that were not noted in the preceding stage and also not noted in sockeye salmon (13).

### Cleavage phase

**Figure 2:**
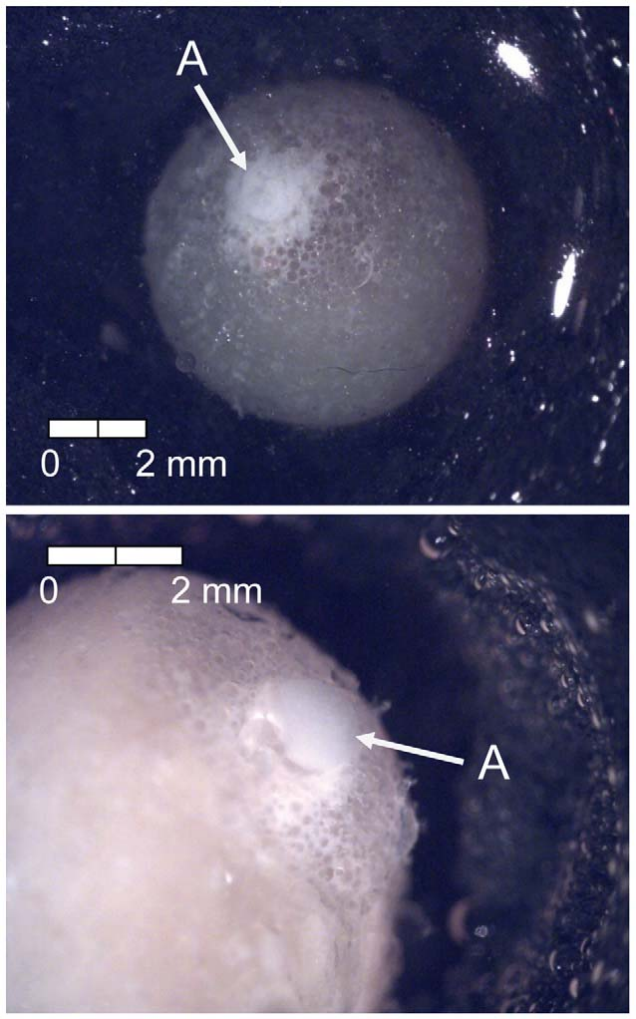
Stage 1 Chinook salmon embryonic development. (A) late morula formed at 34 dd; 3 d (formalin; both, egg capsule removed).

**Figure 3:**
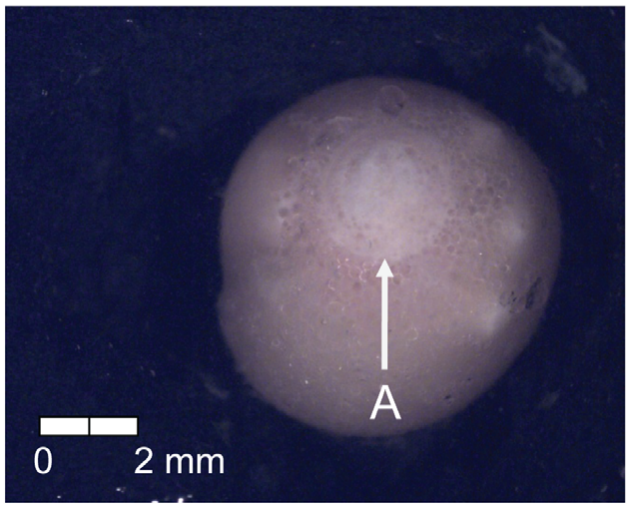
Stage 2 Chinook salmon embryonic development. (A) blastula formation at 66 dd; 6 d (formalin; egg capsule removed).

The first eggs were sampled 34 degree days (dd; 3 d) after fertilization (Table 1). At this time, multiple cell divisions already had occurred and eggs were characterized by the presence of the late morula (Stage 1). The late morula was grainy in appearance (Figure 2) and composed of numerous small cells (i.e., difficult or impossible to quantify). The second sampling event occurred 66 dd (6 d) following fertilization (Stage 2) and was defined by the formation of the blastula from flattening of the edges of the morula (Figure 3).

### Gastrulation phase

**Figure 4:**
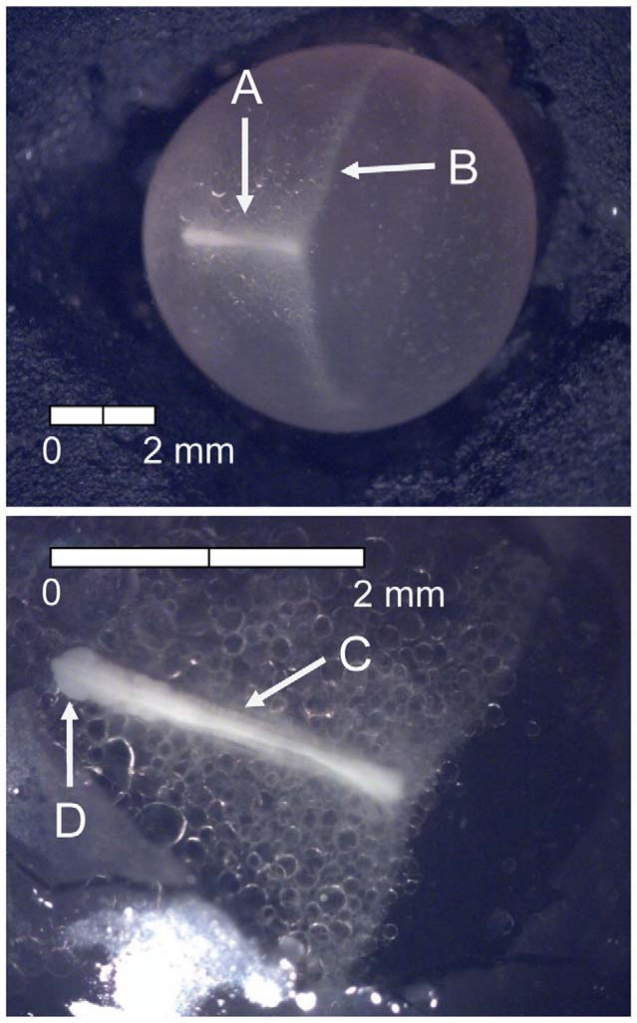
Stage 3 Chinook salmon embryonic development. (A) embryo visible, (B) one-half epiboly, (C) somites, and (D) optic vesicles forming at 96 dd; 9 d (Stockard's; bottom, egg capsule removed).

At 96 dd (9 d) post-fertilization, embryos were visible and epiboly was one-half complete (Stage 3; Figure 4). Additional Stage 3 characteristics included the presence of somites and optic vesicles (Figure 4).

### Organogenesis phase

**Figure 5:**
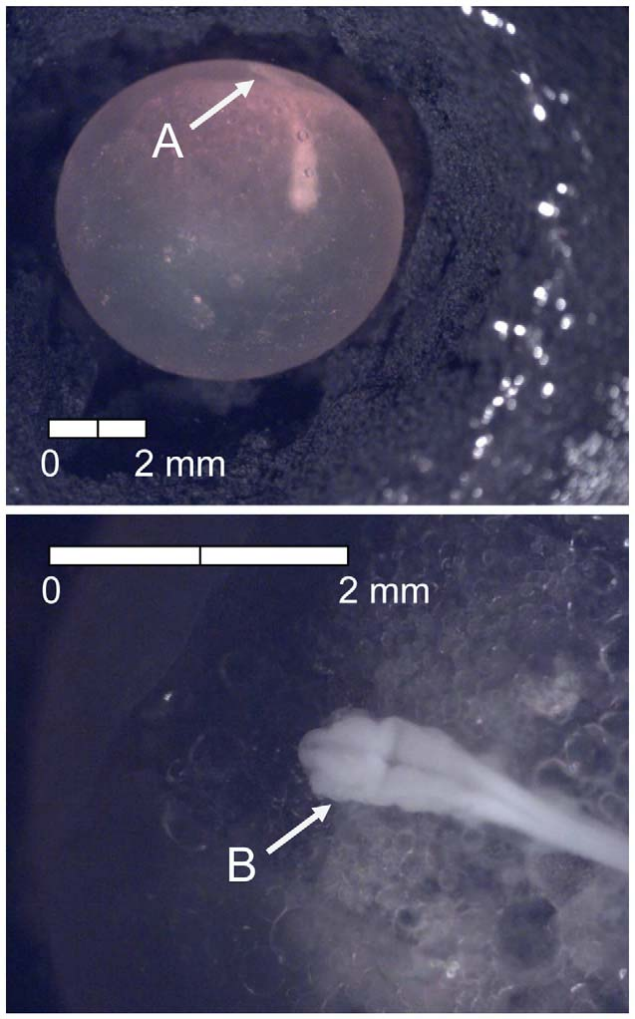
Stage 4 Chinook salmon embryonic development. Epiboly complete, (A) caudal bud free from yolk surface, and (B) lenses visible in eyes at 135 dd; 13 d (Stockard's; bottom, egg capsule removed).

**Figure 6:**
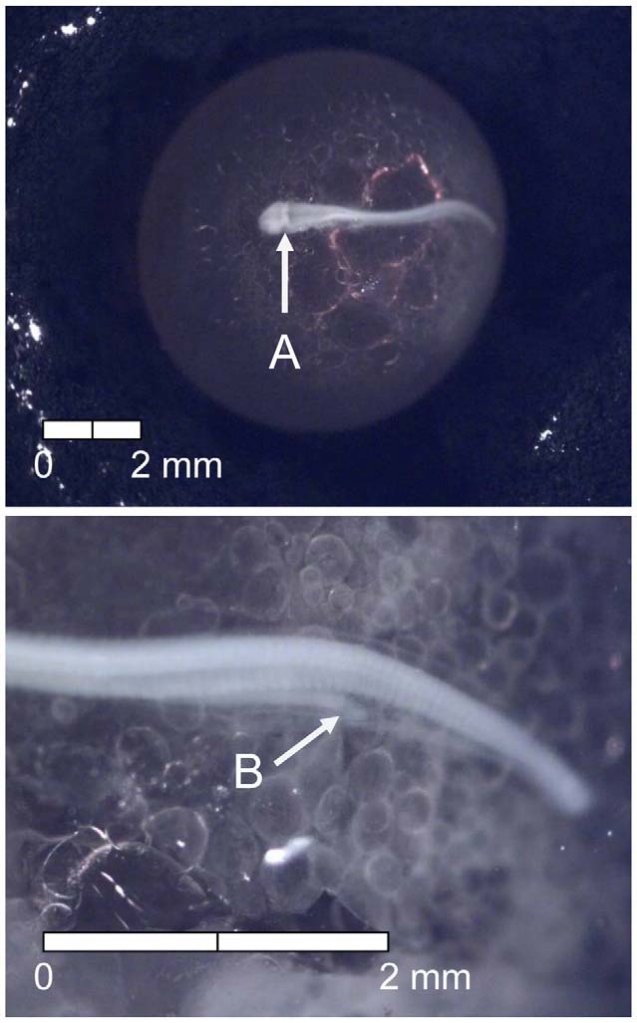
Stage 5 Chinook salmon embryonic development. (A) metencephalon developing and (B) vent visible at 155 dd; 15 d (Stockard's; bottom, egg capsule removed).

**Figure 7:**
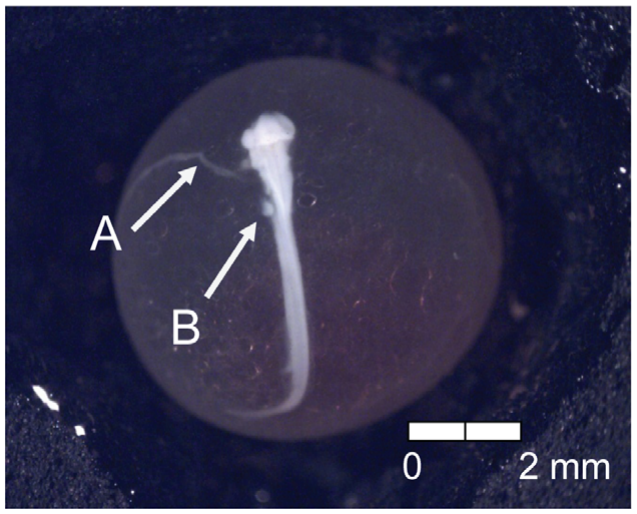
Stage 6 Chinook salmon embryonic development. (A) vitelline vein and (B) pectoral buds visible at 192 dd; 19 d (Stockard's).

**Figure 8:**
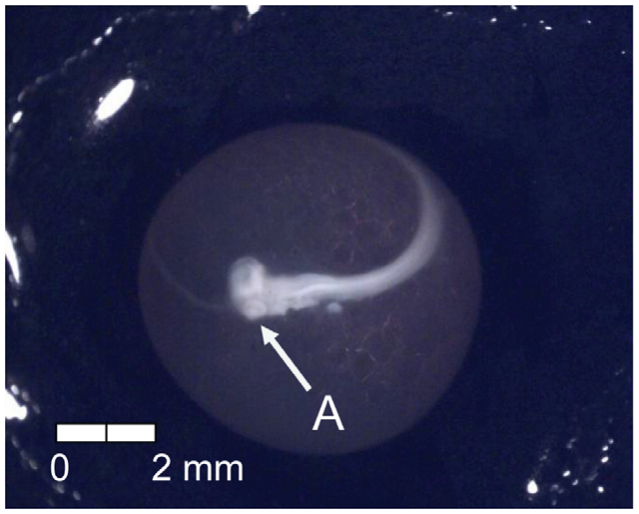
Stage 7 Chinook salmon embryonic development. (A) faint pigmentation in choroid at 211 dd; 21 d (Stockard's).

**Figure 9:**
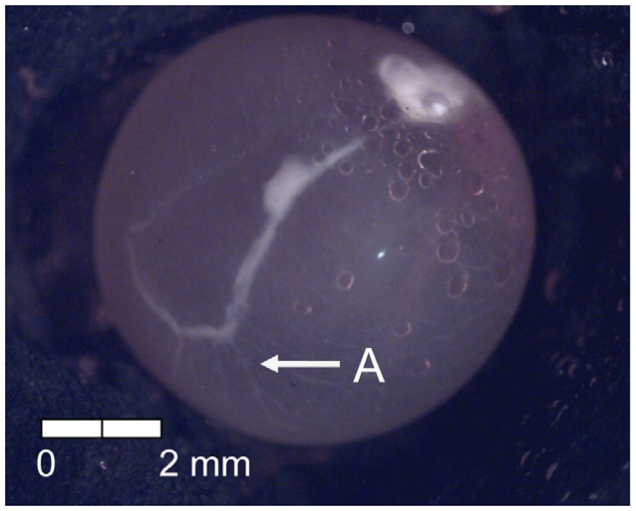
Stage 8 Chinook salmon embryonic development. (A) secondary vascularization branching from vitelline vein at 237 dd; 24 d (Stockard's).

**Figure 10:**
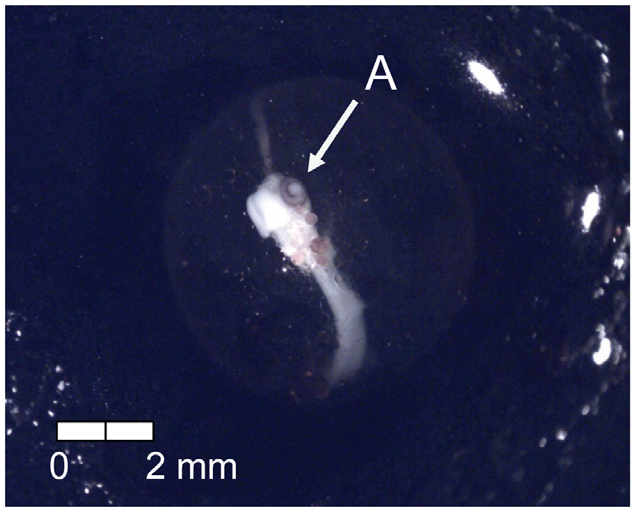
Stage 9 Chinook salmon embryonic development. (A) approximately 50% of choroid pigmented at 261 dd; 27 d (Stockard's; egg capsule removed).

**Figure 11:**
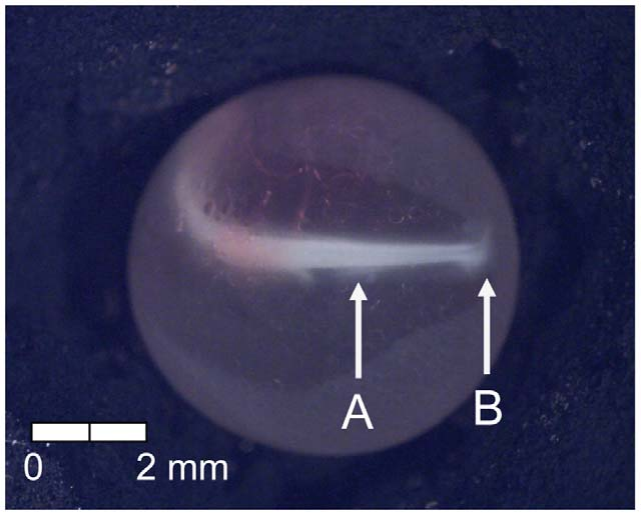
Stage 10 Chinook salmon embryonic development. (A) anal and (B) caudal fins visible as foggy regions in embryonic finfold at 281 dd; 30 d (Stockard's).

**Figure 12:**
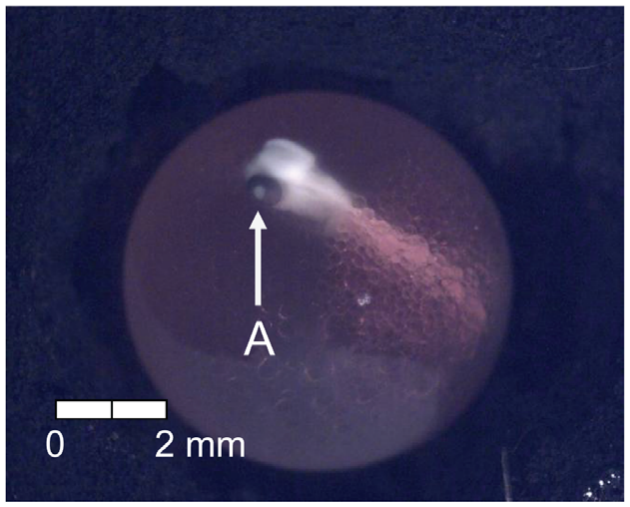
Stage 11 Chinook salmon embryonic development. (A) choroid fully pigmented at 298 dd; 33 d (Stockard's; egg capsule removed).

**Figure 13:**
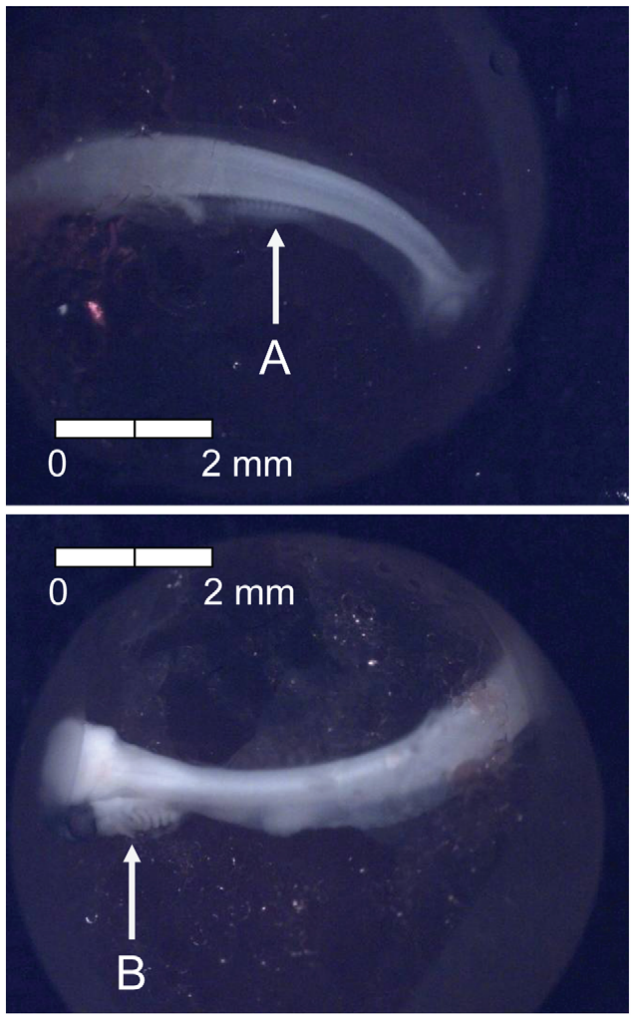
Stage 12 Chinook salmon embryonic development. (A) anal fin rays forming and (B) operculum present but not covering branchial arches at 310 dd; 36 d (Stockard's; bottom, egg capsule removed).

**Figure 14:**
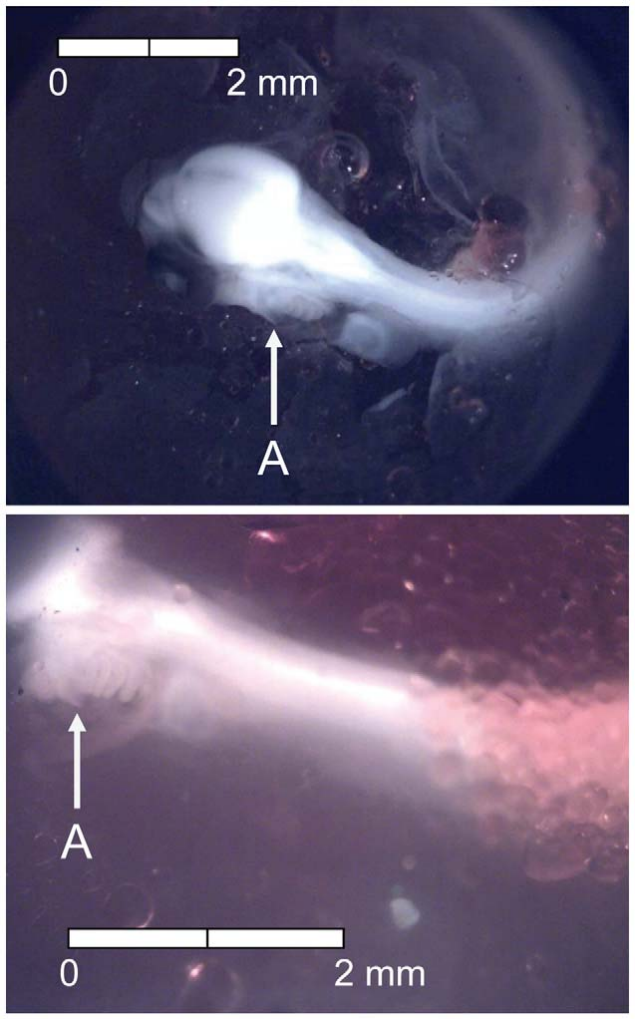
Stage 13 Chinook salmon embryonic development. (A) operculum partially covering first branchial arch at 322 dd; 39 d (Stockard's; bottom, egg capsule removed).

**Figure 15:**
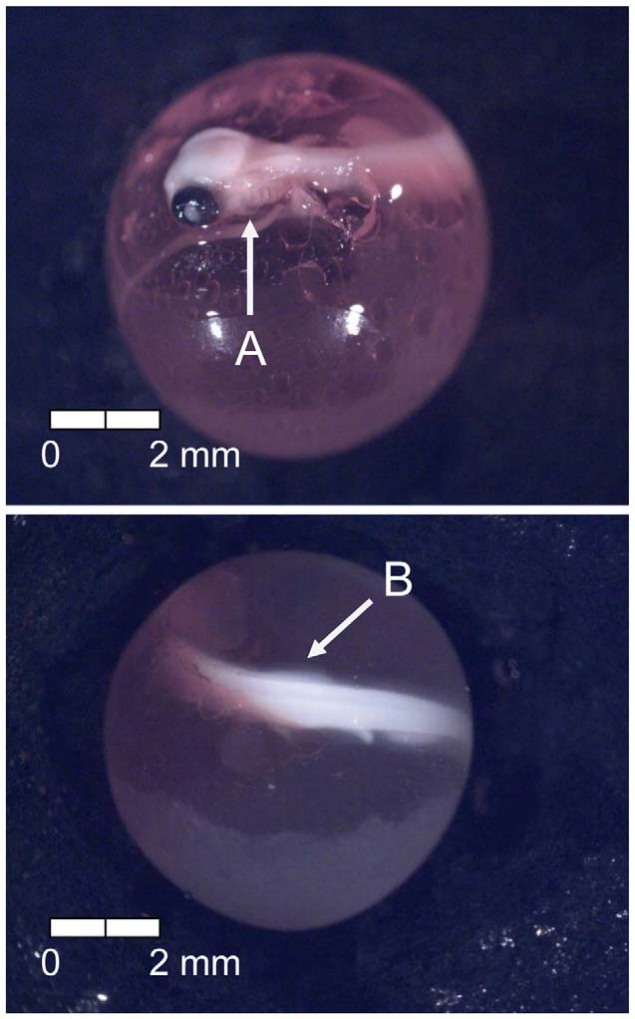
Stage 14 Chinook salmon embryonic development. (A) operculum covers first branchial arch and (B) dorsal fin forming at 336 dd; 42 d (Stockard's; top, egg capsule removed).

**Figure 16:**
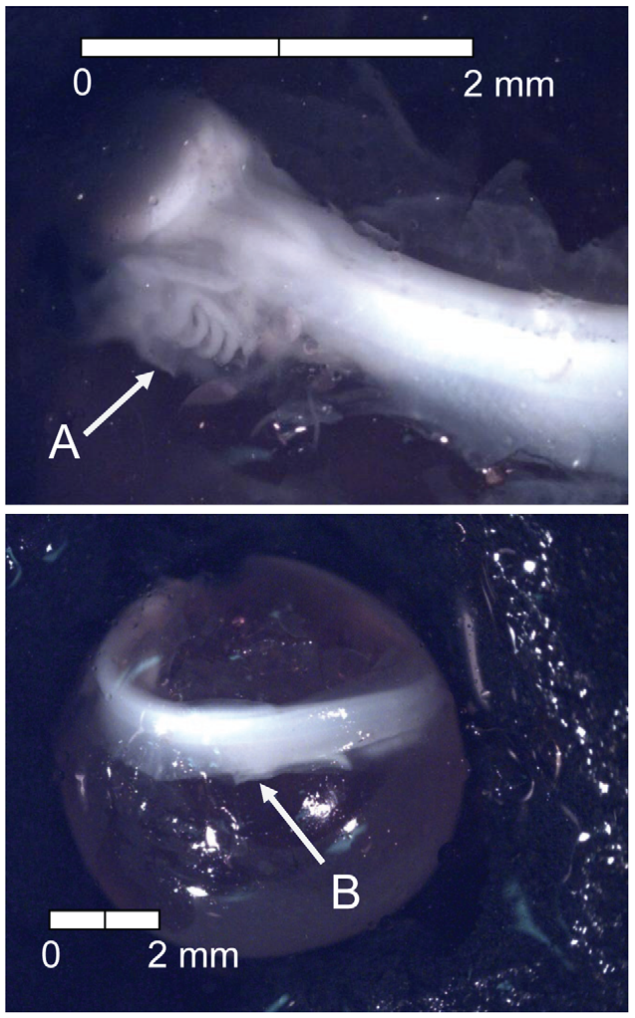
Stage 15 Chinook salmon embryonic development. (A) operculum covers part of second branchial arch and (B) pelvic fin buds visible at 385 dd; 48 d (Stockard's; both, egg capsule removed).

**Figure 17:**
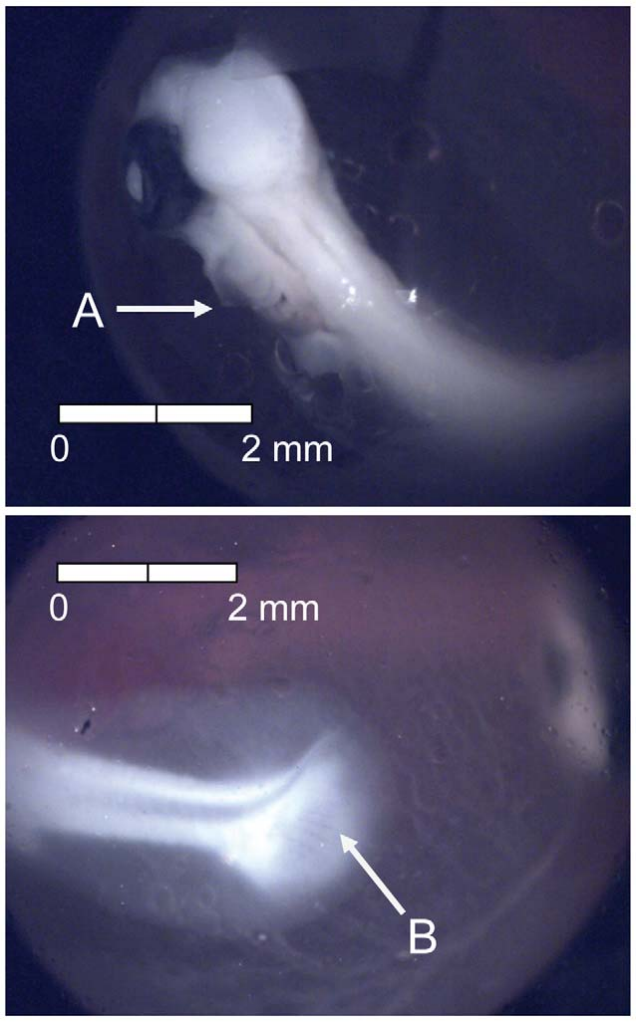
Stage 16 Chinook salmon embryonic development. (A) operculum covers second branchial arch and (B) caudal fin rays visible at 410 dd; 51 d (Stockard's; top, egg capsule removed).

**Figure 18:**
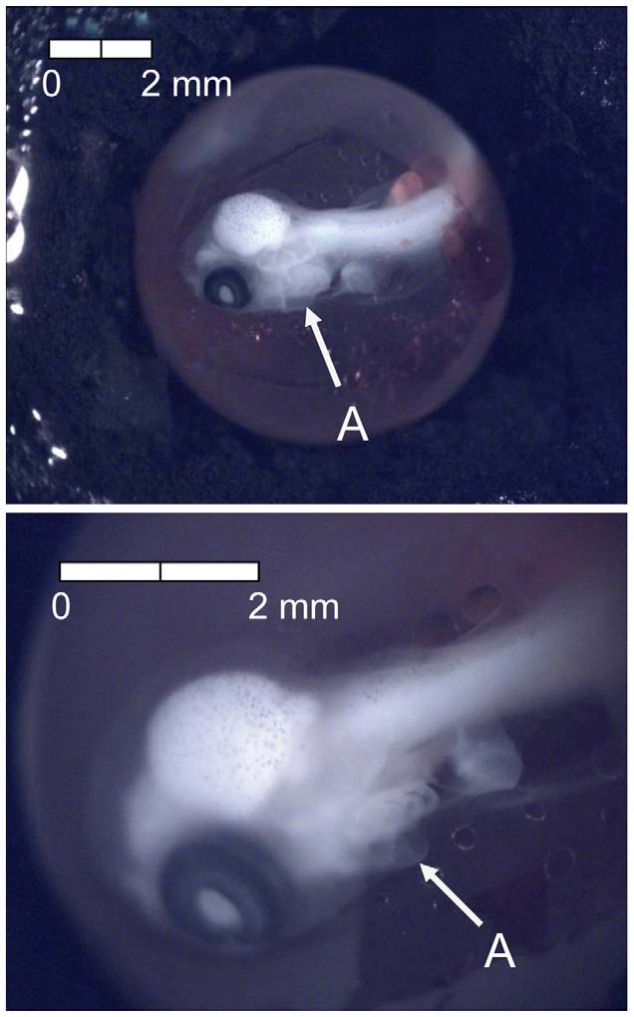
Stage 17 Chinook salmon embryonic development. (A) operculum covers third branchial arch at 435 dd; 54 d (Stockard's; both, egg capsule removed).

**Figure 19:**
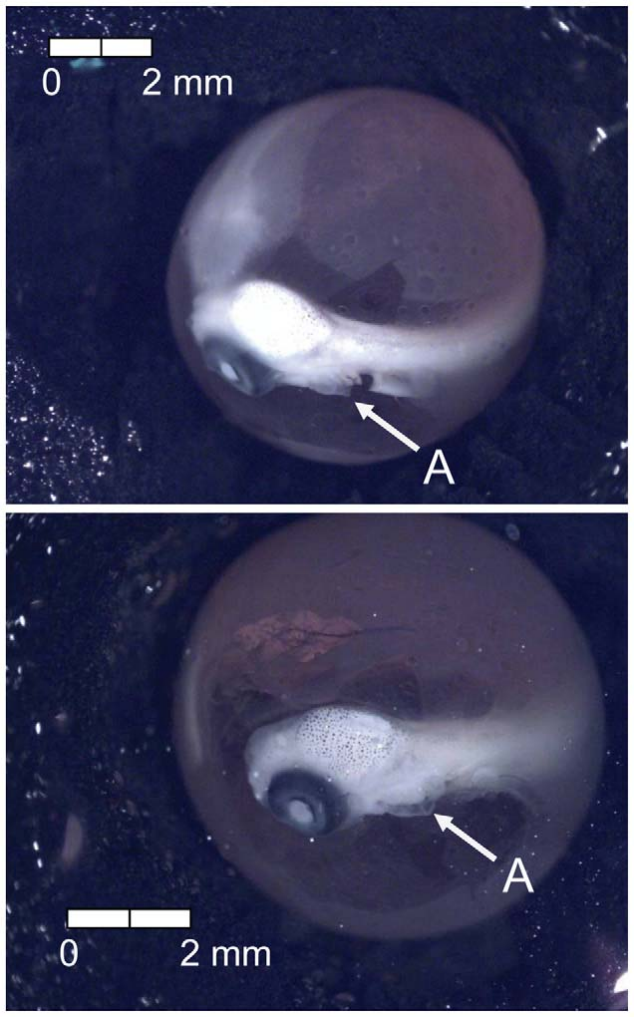
Stage 18 Chinook salmon embryonic development. (A) operculum partially covers fourth branchial arch at 484 dd; 60 d (Stockard's; both, egg capsule removed).

**Figure 20:**
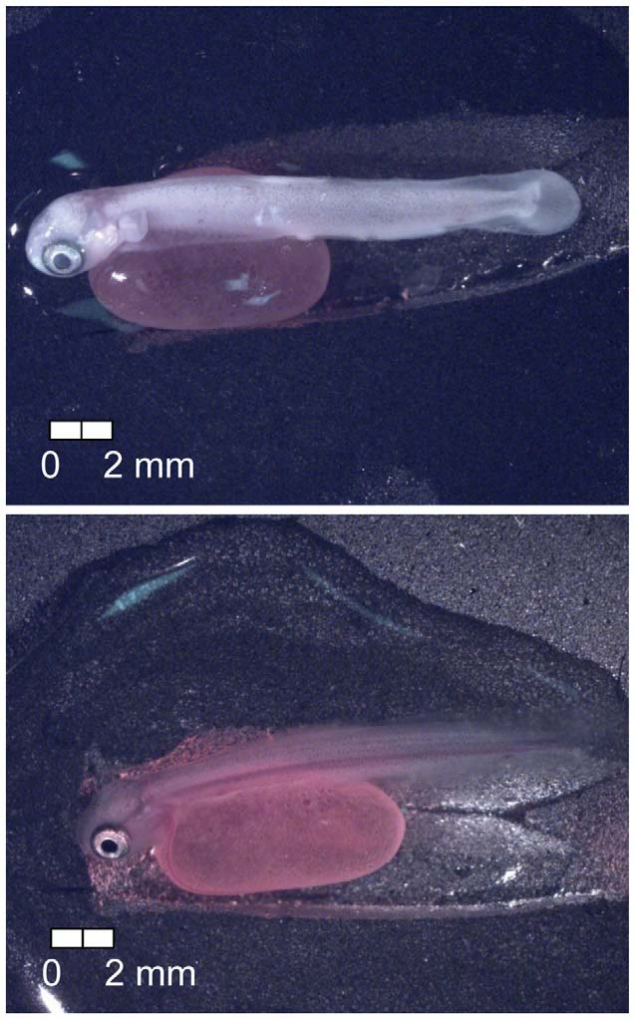
Stage 19 Chinook salmon embryonic development. Fifty percent hatch at 533 dd; 66 d (top, Stockard's; bottom, no fixative).

At 135 dd (13 d) following fertilization, epiboly was complete, the caudal bud of the embryo was free from the surface of the yolk, and lenses were visible in the eyes of the embryos (Stage 4; Figure 5). Stage 5 was characterized by the presence of the metencephalon and anal vent at 155 dd (15 d; Figure 6). The vitelline vein and pectoral fin buds were present by 192 dd (19 d; Stage 6; Figure 7). After 211 dd (21 d), faint pigmentation was visible in the choroid of the eye; this feature characterized Stage 7 (Figure 8). Secondary vascularization branching from the vitelline vein and spreading across the surface of the yolk distinguished Stage 8 (Figure 9). In addition, the head of the embryo was free from the yolk surface by 237 dd (24 d; Figure 9). At 261 dd (27 d) following fertilization, approximately 50% of the choroid of the eye was pigmented (Stage 9; Figure 10). Anal and caudal fins were visible as translucent regions in the embryonic finfold 281 dd (30 d) following fertilization, characterizing Stage 10 (Figure 11). Stage 11 was distinguished by a fully pigmented choroid at 298 dd (33 d; Figure 12). At 310 dd (36 d) after fertilization, anal fin rays and the operculum (which did not yet cover any branchial arches) had formed (Stage 12; Figure 13). The operculum partially covered the first branchial arch in stage 13 (322 dd; 39 d; Figure 14) and totally covered the first branchial arch in stage 14 (336 dd; 42 d; Figure 15). Another identifying feature of Stage 14 was the formation of the dorsal fin in the embryonic finfold (Figure 15). Stage 15 (385 dd; 48 d) was characterized by pelvic fin buds and the operculum partially covering the second branchial arch (Figure 16). The second branchial arch was totally covered by the operculum, and caudal fin rays were visible by 410 dd (51 d) post-fertilization (Stage 16; Figure 17). At 435 dd (54 d), the operculum covered the third branchial arch (Stage 17; Figure 18) and partially covered the fourth branchial arch at 484 dd (60 d; Stage 18; Figure 19). Stage 19 was characterized by 50% hatch of embryos at 533 dd, or 66 d post-fertilization (Figure 20).

## Discussion

The thermal sums model used in this study provided similar estimates of fall Chinook salmon embryonic development rate in water temperatures varying from 3.9–11.7°C (mean = 8°C) to those from several other studies rearing embryos in constant 8°C water temperature. For example, time to reach the same developmental stages (e.g., stages 8, 9, 10, 11, 14, 18, 19, 20, and 21) were less than one degree day different between this study and a similar study [12]. Time to 50% hatch was 533 dd in this study, similar to two estimates of 527.8 dd (Salmonid Egg Incubation Program 2.1 software, Department of Fisheries and Oceans, Nanaimo, BC, Canada) and 560 dd (12). Greater than 97% of variation in hatch time of Chinook salmon reared in water temperatures from 4 to 18°C was explained by degree days [7]. Cumulatively, these results suggest the thermal sums model provides reasonable time estimates for fall Chinook salmon to known embryonic stages, given the range of water temperatures in this study. Further, these results corroborate the assertion that eggs reared at a constant temperature develop at a similar rate to that of eggs reared under ambient temperatures, given the mean daily temperature of the two groups is equal [7].

The use of degree days requires a known mean daily water temperature. However, water temperature was taken only once daily at Priest Rapids Hatchery; thus, diel fluctuations in water temperature and the daily mean water temperature were unknown. Despite infrequent water temperature data, water temperature in the tailrace of Priest Rapids Dam (source of hatchery water) fluctuated less than 1°C daily from October through December 2009 [15]. It is likely that daily water temperature fluctuations in the eggs trays in the hatchery were minimal. Thus, daily water temperature measurements at Priest Rapids Hatchery were assumed to be reasonable approximations of mean daily water temperature.

In this study, egg envelopes from ova fixed in Stockard's solution exhibited better clarity than those fixed in formalin. For example, egg envelopes fixed in Stockard's solution appeared translucent and allowed for identification of certain developmental characteristics within the egg. Egg envelopes from ova fixed in formalin appeared opaque, and removal of the egg envelope was necessary to view developmental characteristics, although only two samples in the present study were fixed in formalin. The clarity of egg envelopes from formalin-preserved samples in which development was greater than Stage 2 is unknown. Despite improved clarity of egg envelopes preserved in Stockard's solution, removal of egg envelopes is recommended for optimum viewing of most samples.

The index of development created for fall Chinook salmon embryos in this study was a pilot effort to relate developmental stages of hatchery-reared Chinook salmon embryos to degree days. Multiple developmental stages observed by Velsen (1980) in sockeye salmon were not noted in this study due to the time interval between sampling events. However, the developmental index provides a reasonable description of timing to known developmental stages of Chinook salmon embryos and was useful in determining developmental stages of wild fall Chinook salmon embryos excavated from redds in the Columbia River. This index should prove useful to other researchers who wish to approximate fertilization dates of Chinook salmon eggs from natural redds, assuming the thermal history of embryos is known.

## Methods

### Ethics Statement

Fertilized eggs were obtained from an external source and reared at our facility. Although avian and other egg-laying vertebrate species develop backbones prior to hatching, OLAW interprets the PHS Policy as applicable to their offspring only after hatching. [From: *ILAR News* 33(4):68–70, Fall, 1991 (http://dels.nas.edu/ilar_n/ilarjournal/33_4/V33_4PublicHealth.shtml)].

Animal facilities were certified by the Association for Assessment and Accreditation of Laboratory Animal Care; animals were handled in accordance with federal guidelines for the care and use of laboratory animals.

Fall Chinook salmon eggs used in this study were fertilized on November 17, 2008 at Priest Rapids Hatchery on the Columbia River near Mattawa, Washington. Eggs were held in egg trays supplied with river water at Priest Rapids Hatchery from fertilization until December 29, 2008, whereupon 300 eggs were transported to the Aquatics Research Laboratory at the Pacific Northwest National Laboratory (PNNL) in Richland, Washington. Eggs were transported to PNNL due to space constraints and management considerations at Priest Rapids Hatchery. River water was not available at PNNL at this time; therefore, eggs were gradually acclimated (i.e., ∼2°C/h) to (mean±SE) 7.9±0.1°C well water over a several hour period. Water temperature remained relatively constant from this point until the end of the study period.

Fifteen eggs were collected 72 h after fertilization and approximately every 72 h thereafter until 50% hatch occurred. Following collection, eggs were immediately fixed in Stockard's solution (85∶6∶5∶4 parts deionized water, glycerin, 37% formaldehyde, and acetic acid, respectively), except the first two samples on November 20 and 23, which were fixed in non-buffered formalin. Stockard's solution was not available until after November 23, therefore non-buffered formalin was used to preserve eggs sampled prior to that time (i.e., November 20 and 23; 13). All egg samples were immersed in their respective fixative for a minimum of three days before viewing.

A minimum of five eggs from each sample were viewed using a Zeiss Stemi 2000-C stereo microscope under varying magnification (10–50×), and digital images were taken with a Megapixel FireWire digital camera attached to the microscope. All eggs were immersed in water, illuminated with four fluorescent gooseneck lights (Zeiss, Inc.; Model KL 1500), and photographed against a black background. For some photographs, egg envelopes were removed with forceps to improve clarity of diagnostic morphological features.

Known embryonic developmental features described for sockeye salmon [13] were used to describe development of all Chinook salmon embryos. Because samples were taken approximately every 72 h, many identifiable features from the cleavage and gastrulation phases of embryonic development were not documented.
